# Safety and efficacy of inspiratory muscle training for preventing adverse outcomes in patients at risk of prolonged hospitalisation

**DOI:** 10.1186/s13063-017-2372-y

**Published:** 2017-12-28

**Authors:** Balbino Rivail Ventura Nepomuceno, Mayana de Sá Barreto, Naniane Cidreira Almeida, Caroline Ferreira Guerreiro, Eveline Xavier-Souza, Mansueto Gomes Neto

**Affiliations:** 1 Medicine and Health, Federal University of Bahia – UFBA, Av. Tancredo Neves, n 1283, Sala 902 – Edf. Ômega – Caminho das Árvores, Salvador, Bahia ZIP 41820-021 Brazil; 2 Department of Biofunção, Institute of Health Sciences - ICS, UFBA, Av. Tancredo Neves, n 1283, Sala 902 – Edf. Ômega – Caminho das Árvores, Salvador, Bahia ZIP 41820-021 Brazil; 3 Reative Physiotherapy Specialist, Av. Tancredo Neves, n 1283, Sala 902 – Edf. Ômega – Caminho das Árvores, Salvador, Bahia ZIP 41820-021 Brazil; 4 Metropolitan Union for Education and Culture, Av. Tancredo Neves, n 1283, Sala 902 – Edf. Ômega – Caminho das Árvores, Salvador, Bahia ZIP 41820-021 Brazil; 5 Research nucleus of the Hospital Roberto Santos, Salvador, Bahia Brazil; 6 Federal University of Bahia – UFBA, School of Medicine, Salvador, Bahia Brazil; 7 Department of Physiotherapy, Institute of Health Sciences-ICS, UFBA, Salvador, BA Brazil

**Keywords:** Respiratory muscles, Muscle weakness, Functionality, Hospitalisation, Length of stay, Mortality

## Abstract

**Background:**

The early institution of inspiratory muscle training on hospitalised patients with no established respiratory deficits could prevent in-hospital adverse outcomes that are directly or indirectly associated to the loss of respiratory muscle mass inherent to a prolonged hospital stay. The objective of the clinical trial is to assess the impact of inspiratory muscle training on hospital inpatient complications.

**Methods:**

This is a double-blind randomised controlled trial. Subjects in the intervention group underwent an inspiratory muscle training loaded with 50% maximum inspiratory pressure twice daily for 4 weeks from study enrolment. Patients were randomly assigned to an inspiratory muscle training group or a sham inspiratory muscle training group. All patients received conventional physiotherapy interventions. Baseline and post-intervention respiratory and peripheral muscle strength, functionality (performance of activities of daily living), length of hospital stay, and death were evaluated. Clinical outcomes were assessed until hospital discharge. This study was approved by the Institutional Hospital Ethics Committee (03/2014).

**Results:**

Thirty-one patients assigned to the inspiratory muscle training group and 34 to the sham inspiratory muscle training group were analysed. Patients in the inspiratory muscle training group had a shorter mean length of hospital stay (35.3 ± 2.7 vs. 41.8 ± 3.5 days, *p* < 0.01) and a lower risk of endotracheal intubation (relative risk (RR) = 0.36; 95% confidence interval (CI) 0.27–0.97; *p* = 0.03) as well as muscle weakness (RR = 0.36; 95% CI 0.19–0.98; *p* = 0.02) and mortality (RR = 0.23; 95% CI 0.2–0.94; *p* = 0.04). The risk of adverse events did not differ significantly between groups.

**Conclusion:**

Inspiratory muscle training was a protective factor against endotracheal intubation, muscle weakness, and mortality.

**Trial registration:**

ClinicalTrials.gov, ID: NCT02459444. Registered on 19 May 2015.

## Background

A prolonged hospital stay is an independent factor of the development of adverse outcomes during the hospitalisation period [1, 2], and it is already known that its devastating functional impacts persist for a 5-year period after hospital discharge [1–3]. Previous studies have demonstrated that decreased mobility, bed confinement, and mechanical ventilation for > 12 h lead to respiratory muscle atrophy and functional deterioration [4]. Decreased mobility makes patients more vulnerable to complications, such as respiratory and peripheral muscle weakness, cardiorespiratory deconditioning, and a decline in physical functionality, all of which are deeply related to increased morbidity and mortality [5–8]. A lack of early mobility was identified by Morris et al. as a predictor of readmission or death within the first year after hospital discharge [9].

Kayambu et al. conducted a systematic review and concluded that physiotherapy in the intensive care unit appears to confer significant benefits including improving quality of life, physical function, and peripheral and respiratory muscle strength; increasing ventilator-free days; and shortening hospital stay. However, none of the included studies used inspiratory muscle training [10].

Inspiratory muscle training is a therapeutic modality that aims to increase inspiratory muscle conditioning via respiratory muscle overload [11, 12]. In a recent systematic review, Gomes-Neto et al. [13] concluded that preoperative inspiratory muscle training significantly improved pulmonary function, shortened length of postoperative hospital stay, and reduced the risk of pulmonary complications. However, postoperative inspiratory muscle training showed significant improvements in pulmonary function only.

Few studies have reported on the effects of the early addition of inspiratory muscle training [14–17], and no published data prove the preventive effect of inspiratory muscle training on prolonged hospitalisation complications. Our hypothesis is that the early institution of inspiratory muscle training on hospitalised patients with no established respiratory deficits could prevent in-hospital adverse outcomes that are directly or indirectly associated to the loss of respiratory muscle mass inherent to a prolonged hospital stay. Thus, the objective of this study was to evaluate the safety and efficacy of inspiratory muscle training for preventing adverse outcomes in patients at risk of prolonged hospitalisation.

The primary objective of the clinical trial is to assess the impact of inspiratory muscle training on hospital inpatient complications. It also aims to describe the safety of early respiratory training in hospitalised patients and to measure inspiratory muscle training responses at respiratory muscle strength, peripheral strength, and functionality of hospitalised patients.

## Methods

This prospective, double-blind (patient and evaluator) randomised controlled trial compared the efficacy of inspiratory muscle training and inspiratory muscle training. The study protocol for this trial was described elsewhere [18]. This study was conducted in accordance with Consolidated Standards of Reporting Trials recommendations [19]. The trial was registered at ClinicalTrials.gov in May 2015 (NCT02459444). The trial was performed at the Roberto Santos General Hospital in Salvador, Bahia, Brazil. This study was approved by the Institutional Hospital Ethics Committee (approval reference number 03/2014). Before enrolment, written informed consent was obtained from participants or their legal guardians.

A sample size of 54 participants was calculated based on a statistical power of 80% to detect a difference of 10% [1, 6] of in-hospital mortality rates between the inspiratory muscle training and sham intervention groups, standard deviation of 3% [6], *α* level of 0.05, and estimated loss of 20%.

The study included every patient admitted to the general ward who met the inclusion criteria. The inclusion criteria were age 18–60 years; admission to the hospital ward; and at least two of the following risk factors for prolonged hospitalisation: two or more comorbidities; sepsis; liver, lung, or kidney diseases; neoplasia; mechanical ventilation; and use of vasopressor or dialysis therapy. Patients with a cognitive disability that made them unable to perform the respiratory training; uncontrolled cardiac arrhythmias; circulatory shock; acute ischaemic heart disease; acute respiratory failure (characterised by a partial pressure of arterial oxygen < 60 mmHg or a partial pressure of arterial carbon dioxide > 50 mmHg); neuromuscular disease or myopathies; or diaphragmatic paresis or paralysis were excluded from the protocol.

After the initial assessment, the patients were randomly assigned to the inspiratory muscle training or sham inspiratory muscle training group in a 1:1 ratio by a blinded investigator with no participant contact. The simple randomisation was performed using RandList® software, in which the participants were allotted to groups by numbered and opaque, sealed envelopes that were opened after the initial assessment of those who met the inclusion criteria. All holding sessions were supervised by a member of a team trained exclusively for a daily intervention. Only this team had access to group allocation and the devices used by each patient. All evaluations were carried out by an evaluator, trained exclusively to evaluate the variables of interest of this research, being the same blind to the allocation in the intervention groups and not participating in the training sessions. As well as no being informed of his allocation in research, the patients only had contact with the device in the moments of sessions, always supervised by the staff trained for interventions.

### Training method

**Fig. 1:**
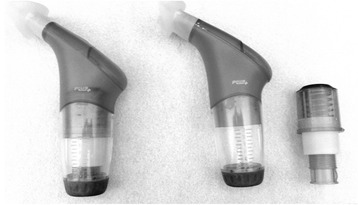
Powerbreathe® Plus calibrated to a high load to the inspiratory muscle training group and unloaded for sham inspiratory muscle training group

Patients in the inspiratory muscle training group performed 4 weeks of inspiratory muscle training using a Powerbreathe® Plus model linear inspiratory resistor device (Fig. 1) loaded with 50% maximal inspiratory pressure. The participants performed one set of 30 breaths twice a day for four consecutive weeks (56 total sessions), according to the protocol used in previous studies [20–24]. The filtre was not used since the devices are for individual use. Heart rate, peripheral oxygen saturation, and the Borg Rating of Perceived Exertion were measured before each session as a safety precaution. Criteria for discontinuation of the exercise included the occurrence of dyspnoea, headache, pain, tachycardia (>20% of the initial heart rate), hypertension (> 20% of the initial blood pressure systolic or diastolic), bronchospasm, dizziness, syncope, or epistaxis [11, 17, 25]. Those receiving the sham inspiratory muscle training performed unloaded exercises with the same training device and method at the same frequency and duration as the inspiratory muscle training group. Both groups performed daily standard physiotherapy that comprised exercises, such as unloaded exercises, muscle stretching, posture, and ambulation, which was instituted as subjects could tolerate it.

### Measurements

The sample characterisation relied on demographical (sex, age), clinical, and functional data. The clinical and functional data comprised cause of hospital admission and illness severity measured by the Acute Physiology and Chronic Health Evaluation (APACHE) score [4]. The Barthel Index and Functional Independence Measure (FIM) were used to assess functional status. The modified Barthel Index is an ordinal scale that provides a total score of 0–100, where higher scores indicate greater independence [26]. The FIM is a scale with an 18-item, seven-level ordinal scale, with 1 indicating dependence and 7 indicating independence [27]. Before the study, each measuring instrument was calibrated according to technical standards.

Peripheral muscle strength was measured according to the Medical Research Council score. The sum score includes strength testing of three arm and three leg muscle groups and results in a score of 0–60 [28, 29]. Muscle weakness was defined as a Medical Research Council score < 48 [4]. Inspiratory muscle strength was assessed by measuring the maximal respiratory pressure using a Wika® CL 1.6 analogue manovacuometer attached to a facial mask and a one-way valve. Five maximal inspiratory and expiratory effort manoeuvers were performed without perioral air leakage and sustained for at least 2 s with values close to each other (10%). The manoeuver with the greatest value was used in the analysis [30, 31]. Respiratory muscle endurance was assessed by maximum voluntary ventilation using a Ferrari® ventilometer. Maximum voluntary ventilation was the largest volume that can be breathed into and out of the lungs during a 10–15-s interval with maximal voluntary effort. [31]

Frequencies of endotracheal intubation, muscle weakness, and death during the intervention period until hospital discharge were recorded. All enrolled patients underwent an intervention protocol for 4 weeks after study inclusion, and their in-hospital adverse outcomes were monitored. All patients who dropped out of the study after at least one intervention, were analysed, considering the intention-to-treat. The occurrence of adverse events, such as dyspnoea, headache, pain, tachycardia, hypertension, bronchospasm, dizziness, syncope, epistaxis, and respiratory failure, during the intervention exercises was noted. Each adverse event was monitored in each intervention session and patients may have presented more than one of these signs or symptoms. [32]

### Data analysis

Continuous variables included mean central tendency and dispersion values, while categorical variables were expressed as absolute and relative frequencies. The Shapiro-Wilk test and central tendency measures were used to determine whether the variables were normally distributed. The analysis of variance test was used to compare mean differences between groups. The statistical analysis was performed using the *X* ^2^ test for categorical variables. Survival was analysed using a Kaplan-Meier survival curve. To assess intervention safety, endotracheal intubation need, muscle weakness, adverse events, and death were assessed by the relative risk (RR) and number needed to treat. *P* values < 0.05 were considered statistically significant. SPSS 21.0 for Windows was used in the data analysis.

## Results

**Fig. 2:**
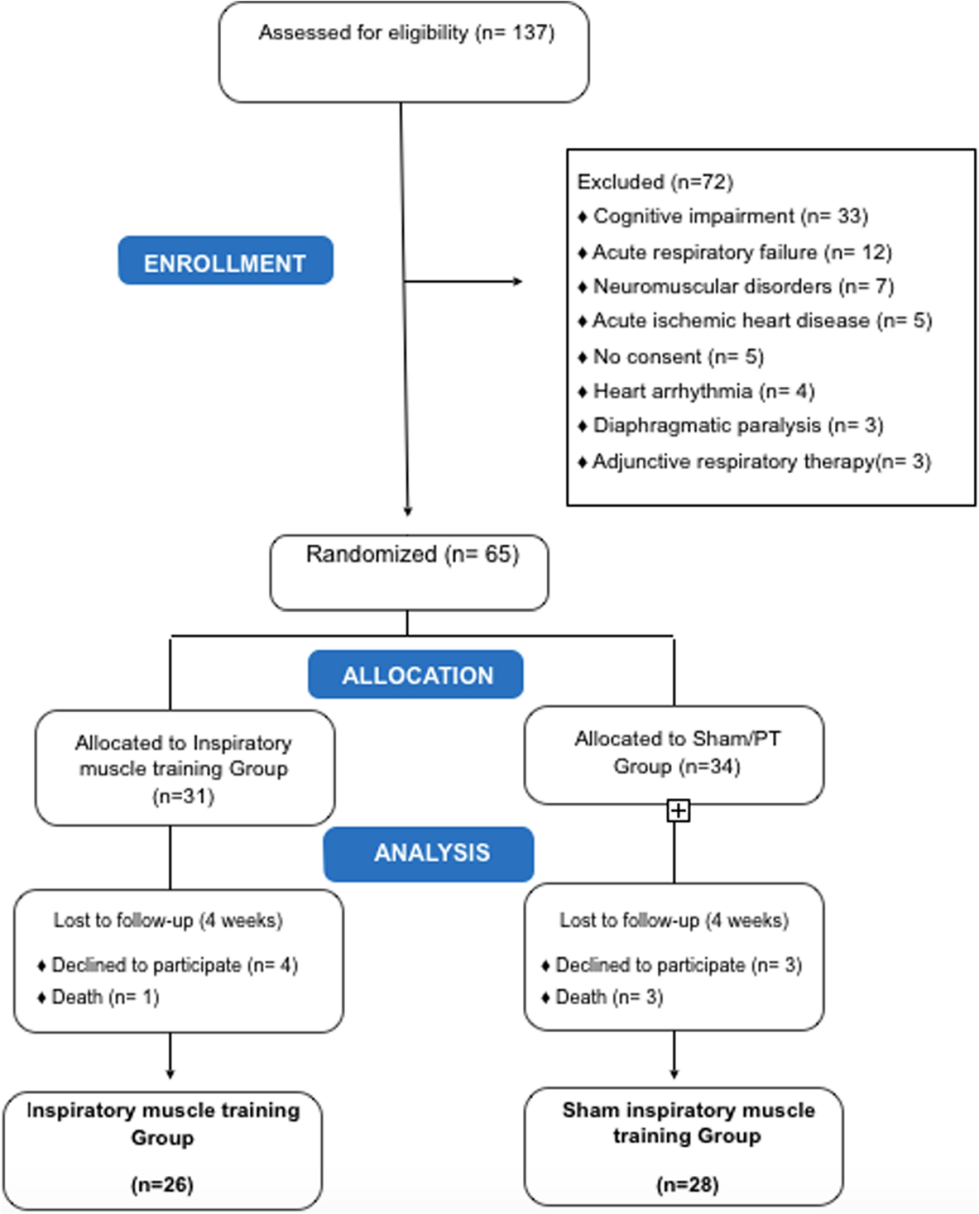
Flow diagram of patient recruitment and follow-up

Of 133 patients admitted to the hospital, 65 were eligible for, and were randomised in, the study (31 in the inspiratory muscle training group, 34 in the sham inspiratory muscle training group. There was a loss of follow-up of five patients for the IMT group and six for the sham group, as shown in Fig. 2. All the randomised patients were included in the final analysis, respecting intention-to-treat.

The sociodemographic and clinical characteristics of the two groups are shown in Table 1. The baseline characteristics were similar in the two groups. The protocol attendance rates did not differ between the two groups (49.4 ± 2.2 vs*.* 48.9 ± 2.0 sessions; *p* = 0.39).

**Table 1:** Distribution of demographic and clinical characteristics by group of intervention in 65 followed patients

	IMT group (*N* = 31)	Sham IMT group (*N* = 34)
Gender:		
Female [*n* (%)]	15 (53.5) = 18 (58.1)	17 (65.3) = 22 (64.7)
Male [*n* (%)]	11 (46.5) = 13 (41.9)	5 (34.7) = 12 (35.3)
Age [mean ± SD]	45.1 ± 1.5	44.0 ± 1.5
Diagnosis [*n* (%)]		
Stroke	5 (19.3) = 5 (16.1)	6 (21.4) = 6 (17.6)
Brain tumour	4 (15.4) = 4 (12.9)	4 (14.3) = 4 (11.8)
Sepsis	4 (15.4) = 4 (12.9)	5 (17.9) = 5 (14.7)
Pneumonia	3 (11.5) = 3 (9.7)	2 (7.1) = 2 (5.8)
Abdominal surgery	3 (11.5) = 3 (9.7)	4 (14.3) = 4 (11.8)
Spinal cord tumour	2 (7.7) = 2 (6.5)	3 (10.7) = 3 (8.8)
Endovascular revascularisation	2 (7.7) = 2 (6.5)	2 (7.1) = 2 (5.8)
Others^a^	3 (11.5) = 3 (9.7)	2 (7.1) = 2 (5.8)
APACHE II [mean ± SD]	21.6 ± 0.4	20.8 ± 0.6
Assiduity to the protocol [mean ± SD]	49.4 ± 2.2	48.9 ± 2.0

*APACHE II* Acute Physiology And Chronic Health Evaluation *IMT group* inspiratory muscle training group, *Sham IMT group* sham inspiratory muscle training group, *SD* standard deviation

^a^Diagnostics with frequency lower than 5% were grouped in Others

Table 2 depicts the patient’s functional status and respiratory variables. The intra-group analysis showed statistically significant improvement in all respiratory and functional parameters for the inspiratory muscle training group only. An intergroup comparative analysis showed significant improvement in the maximal respiratory pressure and functional status for the inspiratory muscle training group (Table 2).

**Table 2:** Baseline and post-intervention data (mean ± SD) on functional and respiratory variables by intervention group in 65 followed patients

	IMT group (*N* = 31)		Sham IMT group (*N* = 34)		*p* value^b^
	Baseline	Fourth week	Baseline	Fourth week	
MIP (cmH_2_O)	− 57.3 ± 27.1	− 87.3 ± 28.2^a^	− 67.6 ± 34.7	− 60.3 ± 25.4	< 0.01
MVV (litres)	11.9 ± 5.2	27.1 ± 15.5^a^	12.3 ± 6.3	12.9 ± 7.6	0.15
MRC	50.0 ± 10.8	55.1 ± 8.0^a^	51.1 ± 9.9	51.3 ± 8.9	0.11
BI	76.5 ± 24.0	87.5 ± 16.5^a^	74.8 ± 22.2	76.2 ± 19.2	0.02
FIM	4.7 ± 2.0	6.0 ± 1.7^a^	4.6 ± 2.0	5.0 ± 1.9	0.06

*IMT group* inspiratory muscle training group, *Sham IMT group* sham inspiratory muscle training group, *MIP* maximal inspiratory pressure, *MVV* maximum voluntary ventilation, *MRC* Medical Research Council, *BI* Barthel Index, *FIM* Functional Independence Measure

^a^Intra-group analysis of baseline and fourth week data (*p* value < 0.05);

^b^Comparing baseline- with fourth-week data between groups

The inspiratory muscle training group had a significantly shorter mean length of hospital stay than the sham inspiratory muscle training group (35.3 ± 2.7 vs*.* 41.8 ± 3.5 days; *p* < 0.01). Furthermore, the inspiratory muscle training protocol was a demonstrated protective factor against endotracheal intubation, muscle weakness, and in-hospital mortality. The prevalence of adverse events during interventions was not significantly different between the two groups (Table 3).

**Table 3:** Data on adverse outcomes and adverse events by intervention group in the followed hospitalised patients

	IMT group (*N* = 31)	Sham IMT group (*N* = 34)	*p* value	RR (CI 95%)	NNT
Hospital LOS (days) [mean ± SD]	35.3 ± 2.7	41.8 ± 3.5	< 0.01	-	-
Patients intubated [*n* (%)]	4 (15.3)	12 (42.8)	0.03	0.36 (0.27–0.97)	3.64
Hospital mortality [*n* (%)]	2 (6.4)	10 (29.4)	0.04	0.23 (0.2–0.94)	8.60
Muscle weakness [*n* (%)]	2 (7.7)	6 (21.4)	0.02	0.36 (0.19–0.98)	7.28
Adverse events^a^ [*n* (%)]	21 (1.6)	22 (1.6)	0.84	1.11 (0.39–3.22)	-
Cephalalgia	6 (0.5)	7 (0.5)	-	-	-
Dyspnoea	6 (0.5)	6 (0.4)	-	-	-
Pain	5 (0.3)	4 (0.3)	-	-	-
Hypertension	4 (0.2)	3 (0.2)	-	-	-
Dizziness	1 (0.1)	2 (0.2)	-	-	-

*CI* confidence interval, *IMT group* inspiratory muscle training group, *Sham IMT group* sham inspiratory muscle training group, *LOS* length of stay, *RR* relative risk, *NNT* number needed to treat, *SD* standard deviation

^a^2655 interventions: 1285 for the inspiratory muscle training group and 1370 for the

sham inspiratory muscle training group

## Discussion

The addition of inspiratory muscle training to physiotherapy was demonstrated safe and resulted in improved inspiratory muscle strength and functional status as well as a shortened length of hospital stay. The inspiratory muscle training was revealed as a protective factor against adverse outcomes.

This randomised controlled trial is relevant because it analyses inspiratory muscle training used as a potential co-adjuvant option for the non-pharmacological prevention and treatment of hospitalised patients. In addition, the eligibility of outcomes, such as hospitalisation complications, endotracheal intubation need, and in-hospital mortality rates, are essential factors for health care and assistance cost evaluations, and these outcomes are potential markers of public health management effectiveness. Beyond its physical and functional benefits, the addition of inspiratory muscle training to standard physiotherapy was a demonstrated protective factor against muscle weakness, promoting a 64% risk reduction for this complication. We calculated that the inspiratory muscle training group must include eight patients to prevent one case of muscle weakness in patients at risk for prolonged hospitalisation. To our knowledge, no other studies in the academic literature have addressed the efficacy of inspiratory muscle training.

Inspiratory muscle training also reduced the need for endotracheal intubation by 64%, resulting in a sample size of four to prevent one intubation. The in-hospital mortality rate was higher in patients in the sham intervention group than in those in the inspiratory muscle training group, with an average of nine required patients to prevent one death. In a recent clinical trial, Wiskemann et al. [33] reported a required six treated patients to prevent one death among oncologic patients who underwent an exercise program from in-hospital admission to 6–8 weeks after discharge. In a case-control study exploring the impact of organised care (including physiotherapy based on early mobilisation) on patients with ischemic stroke assisted at specialised units, Saposnik et al. [34] found that organised care required eight treated patients to prevent 7-day stroke fatality.

The significant increase of 52.4% of maximal inspiratory pressure restates the positive effect of this therapeutic technique on respiratory muscle strengthening of patients with prolonged hospitalisation. The clinical significance of this finding is not restricted to the benefit of the maximal inspiratory pressure increase to near-normal levels, it also reverses the trend for maximal inspiratory pressure decreasing observed in the sham group [6, 29]. Kulkarni et al*.* [14] evaluated the use of different linear training devices for inspiratory muscle training during 2 weeks before major abdominal surgery and noticed that the same linear resistor used in the present study was the only one to show a significant and lasting increase in maximal inspiratory pressure, from 51.5 cmH_2_O to 68.5 cmH_2_O, with no significant reduction in postoperative maximal inspiratory pressure (*p* = 0.36).

De Jonghe et al. [29] identified a strong correlation between decreased maximal inspiratory pressure and reduced peripheral muscle strength. Relying on the principle of muscle reversibility, muscle training benefits the target muscles and their synergists. This theory is supported by Chiang et al. [35] who detected a significant increase in maximal inspiratory pressure from 46.0 to 60.0 cmH_2_O and improved peripheral muscle strength from 50.0 ± 10.8 to 55.1 ± 8.0 kg after a 6-week rehabilitation protocol comprising early mobilisation and inspiratory exercises five times per week.

Previous studies have also reported the association between early rehabilitation, a decreased mechanical ventilation duration and number of hospital readmissions, and shorter hospital length stay, leading to cost savings [36–38]. Although we have not assessed hospitalisation costs, the early inspiratory muscle training addition was demonstrated to be a protective factor against intubation, muscle weakness, and death, which may reduce hospital costs. The results of this study are in accordance with the findings of previous studies on physiotherapy and respiratory exercises in critically ill patients [10, 36, 39]. These findings reinforce the early addition of inspiratory muscle training to standard physiotherapy as a potential tool for fighting complications of prolonged hospitalisation, which can be instituted as routine treatment for patients who fit the profile.

The inspiratory muscle training protocol of our study used a high-intensity load equivalent to 50–60% of the maximal inspiratory pressure. In a published systematic review, Moodie et al. [39] showed that inspiratory muscle training at higher-intensity loads were more effective at improving muscle strength.

This study is a pioneer in the early institution of an inspiratory muscle training protocol for patients at risk of prolonged hospitalisation. The inspiratory muscle training device used herein is a low-cost apparatus that displays a graduated inspiratory resistance, features that facilitate the reproducibility of inspiratory muscle training. In addition, the training protocol used was of relatively short duration, which makes the training a realistic and feasible treatment in the hospital setting.

The promising results presented here encourage the conduction of further investigations in populations with homogeneous clinical characteristics for longer training periods. Nevertheless, study limitations must be stated. Our study was conducted in a single centre, limiting the extrapolation of results for hospital units with different or specialised profiles. However, the use of a representative sample suggests that the benefits identified here may extend to patients who experience prolonged ward hospitalisations in other facilities. In addition, the lack of electrocardiographic monitoring during inspiratory muscle training sessions is a possible limitation because arrhythmia is an adverse event reported in the academic literature for such therapeutic exercises, albeit with low frequency. However, other cardiorespiratory parameters were measured for safety purposes. The chosen parameters presented good reproducibly, low cost, and adequate applicability to patients with conditions of diverse complexity in the hospital environment.

## Conclusion

The early institution of inspiratory muscle training with a loaded linear resistor, associated with standard physiotherapy, is effective at preventing complications due to prolonged hospitalisation and reducing associated in-hospital mortality rates. Its therapeutic use is safe and well-tolerated in the hospital environment, providing respiratory gain and improving functional capacity.

## Abbreviations

APACHE: Acute Physiology and Chronic Health Evaluation Score
FIM: Functional Independence Measure
IMT: Inspiratory muscle training
MIP: Maximal inspiratory pressure
MRC: Medical Research Council
MV: Mechanical ventilation
MVV: Maximum voluntary ventilation
RR: Relative risk

## References

[1] Hermans G, Van Mechelen H, Clerckx B, Vanhullebusch T, Mesotten D, Wilmer A (2014). Acute outcomes and 1-year mortality of intensive care unit-acquired weakness: a cohort study and propensity-matched analysis. Am J Respir Crit Care Med.

[2] Nepomuceno Júnior BRV, Martinez BP, Gomes NM (2014). Impact of hospitalization in an intensive care unit on range of motion of critically ill patients: a pilot study. Rev Bras Ter Intensiva.

[3] Herridge MS, Tansey CM, Matté A, Tomlinson G, Diaz-Granados N, Cooper A (2005). Functional disability 5 years after acute respiratory distress syndrome. N Engl J Med.

[4] Jung B, Moury PH, Mahul M, de Jong A, Galia F, Prades A (2016). Diaphragmatic dysfunction in patients with ICU-acquired weakness and its impact on extubation failure. Intensive Care Med.

[5] Hudson MB, Smuder AJ, Nelson WB, Wiggs MP, Shimkus KL, Fluckey JD (2015). Partial support ventilation and mitochondrial-targeted antioxidants protect against ventilator-induced decreases in diaphragm muscle protein synthesis. PLoS One.

[6] Mukhopadhyay A, Tai BC, See KC, Ng WY, Lim TK, Onsiong S, et al. Risk factors for hospital and long-term mortality of critically ill elderly patients admitted to an intensive care unit. Biomed Res Int. 2014:960575. 10.1155/2014/960575. 10.1155/2014/960575 PMC4280808 25580439

[7] Cameron S, Ball I, Cepinskas G, Choong K, Doherty TJ, Ellis CG, et al. Early mobilization in the critical care unit: a review of adult and pediatric literature. J Crit Care. 2015;30(4):664–72. 10.1016/j.jcrc.2015.03.032. 10.1016/j.jcrc.2015.03.032 25987293

[8] Azevedo PMD, Gomes BP. Effects of early mobilisation in the functional rehabilitation of critically ill patients: a systematic review. J Nurs Ref. 2015;5:129–38. http://dx.doi.org/10.12707/RIV14035.

[9] Morris P, Griffin L, Berry M, Thompson C, Hite D, Winkelman C, Haponik E (2011). Receiving early mobility during an ICU admission is predictor of improved outcomes in acute respiratory failure. Am J Med Sci.

[10] Kayambu G, Boots R, Paratz J (2013). Physical therapy for the critically ill in the ICU: a systematic review and meta-analysis. Crit Care Med.

[11] Charususin N, Gosselink R, Decramer M, McConnell A, Saey D, Maltais F (2013). Inspiratory muscle training protocol for patients with chronic obstructive pulmonary disease (IMTCO study): a multicentre randomised controlled trial. BMJ Open.

[12] Hart N, Sylvester K, Ward S, Cramer D, Moxham J, Polkey MI (2001). Evaluation of an inspiratory muscle trainer in healthy humans. Respir Med.

[13] Gomes Neto M, Martinez BP, Reis HF, Carvalho VO. Pre- and postoperative inspiratory muscle training in patients undergoing cardiac surgery: Systematic review and meta-analysis. Clin Rehabil. 2017;31(4):454-64. 10.1177/0269215516648754. 10.1177/0269215516648754 27154820

[14] Kulkarni SR, Fletcher E, McConnell AK, Poskitt KR, Whyman MR (2010). Pre-operative inspiratory muscle training preserves postoperative inspiratory muscle strength following major abdominal surgery—A randomised pilot study. Ann R Coll Surg Engl.

[15] Plentz RDM, Sbruzzi G, Ribeiro RA, Ferreira JB, Dal LP (2012). Inspiratory muscle training in patients with heart failure: meta-analysis of randomized trials. Arq Bras Cardiol.

[16] Martin AD, Smith BK, Davenport PD, Harman E, Gonzalez-Rothi RJ, Baz M (2011). Inspiratory muscle strength training improves weaning outcome in failure to wean patients: a randomized trial. Crit Care.

[17] Moodie LH, Reeve JC, Vermeulen N, Elkins MR (2011). Inspiratory muscle training to facilitate weaning from mechanical ventilation: protocol for a systematic review. BMC Res Notes..

[18] Nepomuceno Júnior BRV, Gomes NM. Treinamento muscular inspiratório no ambiente hospitalar—protocolo para um ensaio clínico randomizado. Rev Pesqui em Fisioter. 2016;6:158–66. http://dx.doi.org/10.17267/2238-2704rpf.v6i2.896.

[19] Schulz KF, Altman DG, Moher D (2010). CONSORT 2010 statement: updated guidelines for reporting parallel group randomised trials. Int J Surg.

[20] McConnell AK, Lomax M (2006). The influence of inspiratory muscle work history and specific inspiratory muscle training upon human limb muscle fatigue. J Physiol.

[21] Condessa RL, Brauner JS, Saul AL, Baptista M, Silva ACT, Vieira SRR (2013). Inspiratory muscle training did not accelerate weaning from mechanical ventilation but did improve tidal volume and maximal respiratory pressures: a randomised trial. J Physiother.

[22] Caruso P, Carnieli DS, Kagohara KH, Anciães A, Segarra JS, Deheinzelin D (2008). Trend of maximal inspiratory pressure in mechanically ventilated patients: predictors. Clinics.

[23] Paternostro-Sluga T, Grim-Stieger M, Posch M, Schuhfried O, Vacariu G, Mittermaier C (2008). Reliability and validity of the Medical Research Council (MRC) scale and a modified scale for testing muscle strength in patients with radial palsy. J Rehabil Med.

[24] Kleyweg RP, van der Meché FG, Schmitz PI (1991). Interobserver agreement in the assessment of muscle strength and functional abilities in Guillain-Barré syndrome. Muscle Nerve.

[25] Martin AD, Davenport PD, Franceschi AC, Harman E (2002). Use of inspiratory muscle strength training to facilitate ventilator weaning: a series of 10 consecutive patients. Chest.

[26] de Morton NA, Keating JL, Davidson M (2008). Rasch analysis of the Barthel Index in the assessment of hospitalised older patients following admission for an acute medical condition. Arch Phys Med Rehabil..

[27] Stineman MG, Shea JA, Jette A, Tassoni CJ, Ottenbacher KJ, Fiedler R (1996). The Functional Independence Measure: tests of scaling, assumptions, structure, and reliability across 20 diverse impairment categories. Arch Phys Med Rehabil.

[28] De Jonghe B, Sharshar T, Lefaucheur JP (2002). Paresis acquired in the intensive care unit: a prospective multicenter study. JAMA.

[29] De Jonghe B, Bastuji-Garin S, Durand M-C, Malissin I, Rodrigues P, Cerf C (2007). Respiratory weakness is associated with limb weakness and delayed weaning in critical illness. Crit Care Med.

[30] Sclauser Pessoa IM, Franco Parreira V, Fregonezi GA, Sheel AW, Chung F, Reid WD (2014). Reference values for maximal inspiratory pressure: a systematic review. Can Respir J.

[31] Neder JA, Andreoni S, Lerario MC, Nery LE (1999). Reference values for lung function tests: II. maximal respiratory pressures and voluntary ventilation. Braz J Med Biol Res.

[32] Ramos PS, Da Costa da Silva B, da Silva LOG, Araújo CG (2015). Acute hemodynamic and electrocardiographic responses to a session of inspiratory muscle training in cardiopulmonary rehabilitation. Eur J Phys Rehabil Med.

[33] Wiskemann J, Kleindienst N, Kuehl R, Dreger P, Schwerdtfeger R, Bohus M (2015). Effects of physical exercise on survival after allogeneic stem cell transplantation. Int J Cancer.

[34] Saposnik G, Kapral MK, Coutts SB, Fang J, Demchuk AM, Hill MD (2009). Do all age groups benefit from organized inpatient stroke care?. Stroke.

[35] Chiang L-L, Wang L-Y, Wu C-P, Wu H-D, Wu Y-T (2006). Effects of physical training on functional status in patients with prolonged mechanical ventilation. Phys Ther.

[36] Elkins M, Dentice R (2015). Inspiratory muscle training facilitates weaning from mechanical ventilation among patients in the intensive care unit: a systematic review. J Physiother.

[37] Koropolu R, Chandolu S, Needhman DM (2009). Series on early mobilisation of critically Ill patients. Part One: screening and safety issues. ICU Manag.

[38] Malkoç M, Karadibak D, Yildirim Y (2009). The effect of physiotherapy on ventilatory dependency and the length of stay in an intensive care unit. Int J Rehabil Res.

[39] Moodie L, Reeve J, Elkins M (2011). Inspiratory muscle training increases inspiratory muscle strength in patients weaning from mechanical ventilation: a systematic review. J Physiother.

